# Supplementary material for: Unveiling the Bioactive Potential of the Invasive Jellyfish Phyllorhiza punctata Through Integrative Transcriptomic and Proteomic Analyses
Source: Biomolecules. 2025 Aug 4;15(8):1121. doi: 10.3390/biom15081121 (PMC12383608; doi:10.3390/biom15081121)
Supplement: Supplementary file 1 [file biomolecules-15-01121-s001.zip › Suplementary Material/FileS3/PEPFOLD4/XP_065056045_8714/clusters.html]

 PEP-FOLD clusters


Download archive of all models  here   

|  |  |  |
| --- | --- | --- |
| # | file | sOPEP |
| # cluster 1 - Size: 7 |  |  |
|  | XP\_065056045\_8714-model1 | -23.6136 |
|  | XP\_065056045\_8714-model1.1 | -23.385 |
|  | XP\_065056045\_8714-model1.2 | -22.9908 |
|  | XP\_065056045\_8714-model1.3 | -22.8164 |
|  | XP\_065056045\_8714-model1.4 | -22.7214 |
|  | XP\_065056045\_8714-model1.5 | -22.7002 |
|  | XP\_065056045\_8714-model1.6 | -22.0403 |
| # cluster 2 - Size: 30 |  |  |
|  | XP\_065056045\_8714-model2 | -23.3747 |
|  | XP\_065056045\_8714-model2.1 | -23.328 |
|  | XP\_065056045\_8714-model2.2 | -23.2431 |
|  | XP\_065056045\_8714-model2.3 | -23.2077 |
|  | XP\_065056045\_8714-model2.4 | -23.0989 |
|  | XP\_065056045\_8714-model2.5 | -23.09 |
|  | XP\_065056045\_8714-model2.6 | -22.8817 |
|  | XP\_065056045\_8714-model2.7 | -22.8067 |
|  | XP\_065056045\_8714-model2.8 | -22.7433 |
|  | XP\_065056045\_8714-model2.9 | -22.6843 |
|  | XP\_065056045\_8714-model2.10 | -22.622 |
|  | XP\_065056045\_8714-model2.11 | -22.6036 |
|  | XP\_065056045\_8714-model2.12 | -22.5726 |
|  | XP\_065056045\_8714-model2.13 | -22.5239 |
|  | XP\_065056045\_8714-model2.14 | -22.5036 |
|  | XP\_065056045\_8714-model2.15 | -22.4829 |
|  | XP\_065056045\_8714-model2.16 | -22.4311 |
|  | XP\_065056045\_8714-model2.17 | -22.3404 |
|  | XP\_065056045\_8714-model2.18 | -22.3101 |
|  | XP\_065056045\_8714-model2.19 | -22.2687 |
|  | XP\_065056045\_8714-model2.20 | -22.2268 |
|  | XP\_065056045\_8714-model2.21 | -22.0425 |
|  | XP\_065056045\_8714-model2.22 | -22.0281 |
|  | XP\_065056045\_8714-model2.23 | -21.878 |
|  | XP\_065056045\_8714-model2.24 | -21.8603 |
|  | XP\_065056045\_8714-model2.25 | -21.6911 |
|  | XP\_065056045\_8714-model2.26 | -21.5965 |
|  | XP\_065056045\_8714-model2.27 | -21.421 |
|  | XP\_065056045\_8714-model2.28 | -20.7955 |
|  | XP\_065056045\_8714-model2.29 | -20.1166 |
| # cluster 3 - Size: 2 |  |  |
|  | XP\_065056045\_8714-model3 | -22.9741 |
|  | XP\_065056045\_8714-model3.1 | -20.6153 |
| # cluster 4 - Size: 5 |  |  |
|  | XP\_065056045\_8714-model4 | -22.8639 |
|  | XP\_065056045\_8714-model4.1 | -22.6897 |
|  | XP\_065056045\_8714-model4.2 | -22.5242 |
|  | XP\_065056045\_8714-model4.3 | -22.341 |
|  | XP\_065056045\_8714-model4.4 | -22.0619 |
| # cluster 5 - Size: 17 |  |  |
|  | XP\_065056045\_8714-model5 | -22.8484 |
|  | XP\_065056045\_8714-model5.1 | -22.6942 |
|  | XP\_065056045\_8714-model5.2 | -22.541 |
|  | XP\_065056045\_8714-model5.3 | -22.336 |
|  | XP\_065056045\_8714-model5.4 | -22.2316 |
|  | XP\_065056045\_8714-model5.5 | -22.1063 |
|  | XP\_065056045\_8714-model5.6 | -22.0814 |
|  | XP\_065056045\_8714-model5.7 | -22.0495 |
|  | XP\_065056045\_8714-model5.8 | -21.8988 |
|  | XP\_065056045\_8714-model5.9 | -21.8673 |
|  | XP\_065056045\_8714-model5.10 | -21.8261 |
|  | XP\_065056045\_8714-model5.11 | -21.7335 |
|  | XP\_065056045\_8714-model5.12 | -21.6677 |
|  | XP\_065056045\_8714-model5.13 | -21.6167 |
|  | XP\_065056045\_8714-model5.14 | -21.5897 |
|  | XP\_065056045\_8714-model5.15 | -21.3239 |
|  | XP\_065056045\_8714-model5.16 | -21.2332 |
| # cluster 6 - Size: 3 |  |  |
|  | XP\_065056045\_8714-model6 | -22.8476 |
|  | XP\_065056045\_8714-model6.1 | -22.712 |
|  | XP\_065056045\_8714-model6.2 | -20.6406 |
| # cluster 7 - Size: 1 |  |  |
|  | XP\_065056045\_8714-model7 | -22.661 |
| # cluster 8 - Size: 14 |  |  |
|  | XP\_065056045\_8714-model8 | -22.6458 |
|  | XP\_065056045\_8714-model8.1 | -22.1302 |
|  | XP\_065056045\_8714-model8.2 | -22.0172 |
|  | XP\_065056045\_8714-model8.3 | -21.861 |
|  | XP\_065056045\_8714-model8.4 | -21.7716 |
|  | XP\_065056045\_8714-model8.5 | -21.6902 |
|  | XP\_065056045\_8714-model8.6 | -21.6537 |
|  | XP\_065056045\_8714-model8.7 | -21.6212 |
|  | XP\_065056045\_8714-model8.8 | -21.5621 |
|  | XP\_065056045\_8714-model8.9 | -21.5437 |
|  | XP\_065056045\_8714-model8.10 | -21.0193 |
|  | XP\_065056045\_8714-model8.11 | -20.91 |
|  | XP\_065056045\_8714-model8.12 | -20.8709 |
|  | XP\_065056045\_8714-model8.13 | -20.3614 |
| # cluster 9 - Size: 4 |  |  |
|  | XP\_065056045\_8714-model9 | -22.5216 |
|  | XP\_065056045\_8714-model9.1 | -21.8475 |
|  | XP\_065056045\_8714-model9.2 | -21.6812 |
|  | XP\_065056045\_8714-model9.3 | -21.3947 |
| # cluster 10 - Size: 5 |  |  |
|  | XP\_065056045\_8714-model10 | -22.268 |
|  | XP\_065056045\_8714-model10.1 | -21.0237 |
|  | XP\_065056045\_8714-model10.2 | -20.9627 |
|  | XP\_065056045\_8714-model10.3 | -20.7682 |
|  | XP\_065056045\_8714-model10.4 | -20.5381 |
| # cluster 11 - Size: 1 |  |  |
|  | XP\_065056045\_8714-model11 | -22.1763 |
| # cluster 12 - Size: 2 |  |  |
|  | XP\_065056045\_8714-model12 | -21.7873 |
|  | XP\_065056045\_8714-model12.1 | -20.9411 |
| # cluster 13 - Size: 1 |  |  |
|  | XP\_065056045\_8714-model13 | -21.7129 |
| # cluster 14 - Size: 3 |  |  |
|  | XP\_065056045\_8714-model14 | -21.56 |
|  | XP\_065056045\_8714-model14.1 | -21.2049 |
|  | XP\_065056045\_8714-model14.2 | -20.9082 |
| # cluster 15 - Size: 1 |  |  |
|  | XP\_065056045\_8714-model15 | -21.5267 |
| # cluster 16 - Size: 1 |  |  |
|  | XP\_065056045\_8714-model16 | -21.1886 |
| # cluster 17 - Size: 1 |  |  |
|  | XP\_065056045\_8714-model17 | -20.9287 |
| # cluster 18 - Size: 2 |  |  |
|  | XP\_065056045\_8714-model18 | -20.6768 |
|  | XP\_065056045\_8714-model18.1 | -20.0422 |
|  |  |  |
